# Supplementary material for: Genetic characterization of candidate ecdysteroid kinases in Drosophila melanogaster
Source: G3 (Bethesda). 2024 Aug 29;14(11):jkae204. doi: 10.1093/g3journal/jkae204 (PMC12117438; doi:10.1093/g3journal/jkae204)
Supplement: jkae204_Supplementary_Data [file jkae204_Supplementary_Data.zip › Supplemental_Methods_and_Figures_G3-2024-405186.docx]

**Supplementary Materials**

**Supplementary Methods**

**Fly crossing for genotype creation**

*w^1118^*; *Kr^If-1^*/CyO *actGFP*; *Sb^1^*/TM3 *actGFP* *Ser^1^* (also known as *w^1118^*-DB^GFP^) was made by routine crosses, starting with separately crossing BL4534 and BL36320 to *w^1118^*; *Kr^IF-1^*/*CyO*; *Sb^1^*/*TM6B Antp^Hu^ Tb^1^* (herein *w^1118^*-DB) until the desired genotype was reached. FM7j; 25709; 25709 flies were made by routine crosses, starting with separately crossing BL6418 and BL25709 with *w^1118^*-DB, until the desired genotype was reached. FM7i, *actGFP*/C(1)DX *y^1^* *f^1^*; 25709; 25709 flies were made by routine crosses, starting with separately crossing BL4559 and BL25709 with *w^1118^*-DB, until the desired genotype was reached. FM7j;; *Sb^1^*/TM6B *Antp^Hu^* *Tb^1^* flies were made by routine crosses, starting with BL6418 females and *w^1118^*-DB males, until the desired genotype was reached. The FM7c balancer was crossed out from *y^1^* *w^*^* *P{lacW}Fas2G0032* *P{neoFRT}19A*/FM7c; *P{ey-FLP.N}5* using *w^1118^*-DB and then crossed to *w^1118^*. *pkm^2^*/FM7c;; *Wall^1^* flies were made by routine crosses, starting with crossing *pkm^2^*/FM7i *actGFP* females and FM7c/Y males, *w^1118^*-DB females and FM7c/Y males, and *w^1118^*-DB females and *Wall^1^* males; the presence of each deletion allele in the final stock was confirmed by PCR. The chr3 UAS-*Cas9* construct was crossed out of BL67083 by routine crosses involving *w^1118^*-DB. UAS-*Wall^pCFD6^*/CyO *actGFP*; UAS-*Cas9* flies were made by routine crosses, starting with separately crossing UAS-*Wall^pCFD6^* and UAS-*Cas9* to *w^1118^*-DB^GFP^, until the desired genotype was reached. UAS-*pkm^pCFD6^*; UAS-*Cas9*/TM3 *actGFP*, *Ser^1^* flies were made by routine crosses, starting with separately crossing UAS-*pkm^pCFD6^* and UAS-*Cas9* to *w^1118^*-DB^GFP^, until the desired genotype was reached. UAS-*Dcr2*/CyO *actGFP*; *tub-GAL4*/TM3 *actGFP* *Ser^1^* was made by routine crosses, starting with UAS-*Dcr2*/CyO; *tub-GAL4*/TM6B and *w^1118^*-DB^GFP^, and selecting the appropriate genotypes. *tub-GAL80^ts^*; *Sb^1^*/TM6B *Antp^Hu^* *Tb^1^* was made by routine crosses, starting with *w^*^*; *tub-GAL80^ts^*; TM2/TM6B males and *w^1118^*-DB females, until the desired genotype was reached. *tub-GAL80^ts^*; UAS-*Wall^EY^* flies were made by routine crosses, starting with BL23106 males and *w^1118^*-DB females, and then crossing to *tub-GAL80^ts^*; *Sb^1^*/TM6B *Antp^Hu^* *Tb^1^*, until the desired genotype was reached. *tub-GAL80^ts^*; *da-GAL4* flies were made by routine crosses, starting with *da-GAL4* males and *w^1118^*-DB females, and then crossing to *tub-GAL80^ts^*; *Sb^1^*/TM6B *Antp^Hu^* *Tb^1^*, until the desired genotype was reached. *Cyp18a1^1^*/FM7i *actGFP*; UAS-*Wall^pU^* flies were made by routine crosses, starting with crossing FM7j;; *Sb^1^*/TM6B *Antp^Hu^* *Tb^1^* females to UAS-*Wall^pU^* males, and *Cyp18a1^1^*/FM7i *actGFP* females to FM7j/Y;; *Sb^1^*/TM6B *Antp^Hu^* *Tb^1^* males, until the desired genotype was reached. *c204-GAL4*/TM3 *actGFP* *Ser^1^* flies were made by crossing BL3751 and BL4534 and selecting the appropriate genotypes. *w^1118^*; *Kr^If-1^*/CyO; *nos-GAL4* UAS-*Cas9* was made as previously described (Scanlan *et al.* 2022).

*pkm^2^* *w^-^*; KK*^pkm^* *w^+mC^*; + lines were made by crossing *pkm^2^* *w^-^*; +; + females to *w^-^*; KK*^pkm^* *w^+mC^*; + males (VL106503), then crossing F1 males (*pkm^2^* *w^-^*/Y; KK*^pkm^* *w^+mC^*/+; +) back to *pkm^2^* *w^-^*; +; + females. Red-eyed male and female F2 individuals (*pkm^2^* *w^-^*; KK*^pkm^* *w^+mC^*/+) were crossed together and pairs of single male and female red-eyed F3 offspring (either *pkm^2^* *w^-^*; KK*^pkm^* *w^+mC^*/+; + or *pkm^2^* *w^-^*; KK*^pkm^* *w^+mC^*; + which are virtually phenotypically identical as a single copy of *w^+mC^* results in a near-wild-type eye colour) were crossed to establish separate lines that were either homozygous for KK*^pkm^* *w^+mC^* or segregating a wild-type 2^nd^ chromosome. DNA from the parents of each line was individually prepared using the single fly DNA extraction protocol (see below) and a genomic reverse primer (NC_R1; Table S2) was used, along with the NC_Genomic_F and pKC26_R primers from Green et al*.* (2014), to genotype the KK-RNAi library insertion site (VIE-260B-2) on the 2^nd^ chromosome in a three-primer PCR using GoTaq Green Master Mix—cycling conditions: 3 min 95 ºC initial denaturation, followed by 35 cycles of 30 sec 95 ºC denaturation, 30 sec 60 ºC annealing and 45 sec 72 ºC extension, then 5 min 72 ºC final extension. NC_Genomic_F and NC_R1 produce a 724 bp amplicon from wild-type chromosomes, while NC_Genomic_F and pKC26_R produce a ~600 bp amplicon from KK*^pkm^* *w^+mC^* chromosomes (the large NC_Genomic_F and NC_R1 amplicon from KK*^pkm^* *w^+mC^* chromosomes fails to amplify with a 45 sec extension). PCR reactions were run on a 1.5% agarose gel for 20 min at 300V, and the presence of a single 724 bp band (and absence of a ~600 bp band) from both parents indicated the resultant line was completely homozygous for KK*^pkm^* *w^+mC^*. DNA from VL106503 flies was used as a homozygous KK*^pkm^* *w^+mC^* control, and DNA from a *Wall^1^* fly was used as a homozygous wild-type 2^nd^ chromosome control.

**Single fly DNA extraction**

Single flies were placed in a 200 µL PCR tube and crushed for 10 sec with a 100 µL pipette tip filled with 50 µL of squishing buffer (10 mM Tris.HCl (pH = 8.2), 1 mM EDTA, 25 mM NaCL, 200 µg/mL Proteinase K), then the buffer liquid was completely voided into the tube. Tubes were incubated at 37 ºC for 30 min then 85 ºC for 5 min. DNA preps were stored at -20 ºC.

**Agar juice plates**

For 1 L media (100 plates): 12.5 g sucrose and two lots of 10 g agar were each separately dissolved in 250 mL dH_2_O (750 mL total) in the microwave, and added to 250 mL of boiled apple juice (final concentrations: 2% w/v agar, 3.125% w/v sucrose, 25% v/v apple juice). 10 mL of media was aliquoted into 60 mm plates and stored at 4 ºC until use.

**Supplementary Tables**

**Table S1.** GAL4 driver lines used in this study.

**Table S2.** Primer sequences used in this study. All oligonucleotides were synthesised by Integrated DNA Technologies (IDT).

**Table S3.** Summary of knockout (germline and somatic CRISPR) and knockdown (RNAi) phenotypes of both *Wall* and *pkm*, derived from Figs. 1, 2, S4 and S5.

**Table S4.** Phenotypic descriptions of misexpression of *Cyp18a1* and *Wall* with various GAL4 drivers (see Table S1).

**Supplementary Figures**

**
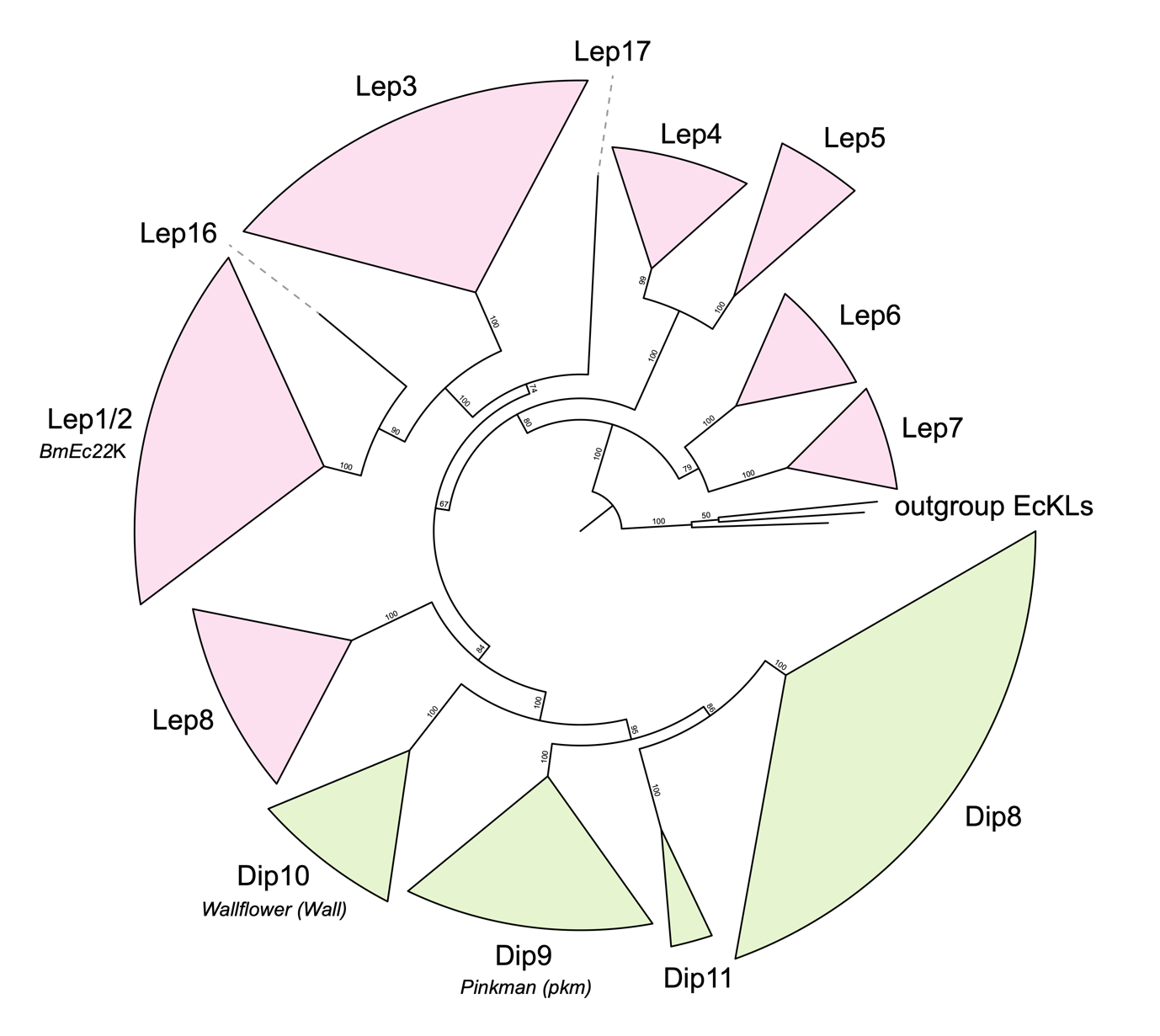
**

**Figure S1.** Phylogenetic tree of EcKLs in subfamily A from the orders Diptera (green) and Lepidoptera (pink), collapsed to the order-ancestral clades defined in Scanlan & Robin (2024) , with the locations of the *BmEc22K*, *Wall* and *pkm* genes noted. The Lep1 and Lep2 clades are not well resolved on this tree and have been collapsed together. The tree was generated as described in Scanlan & Robin (2024). Branch numbers are ultrafast bootstrap support values from UFBoot2, where values of 95 or above are considered reliable (Hoang *et al.* 2018). Tree is rooted with EcKLs from outside of the A subfamily.


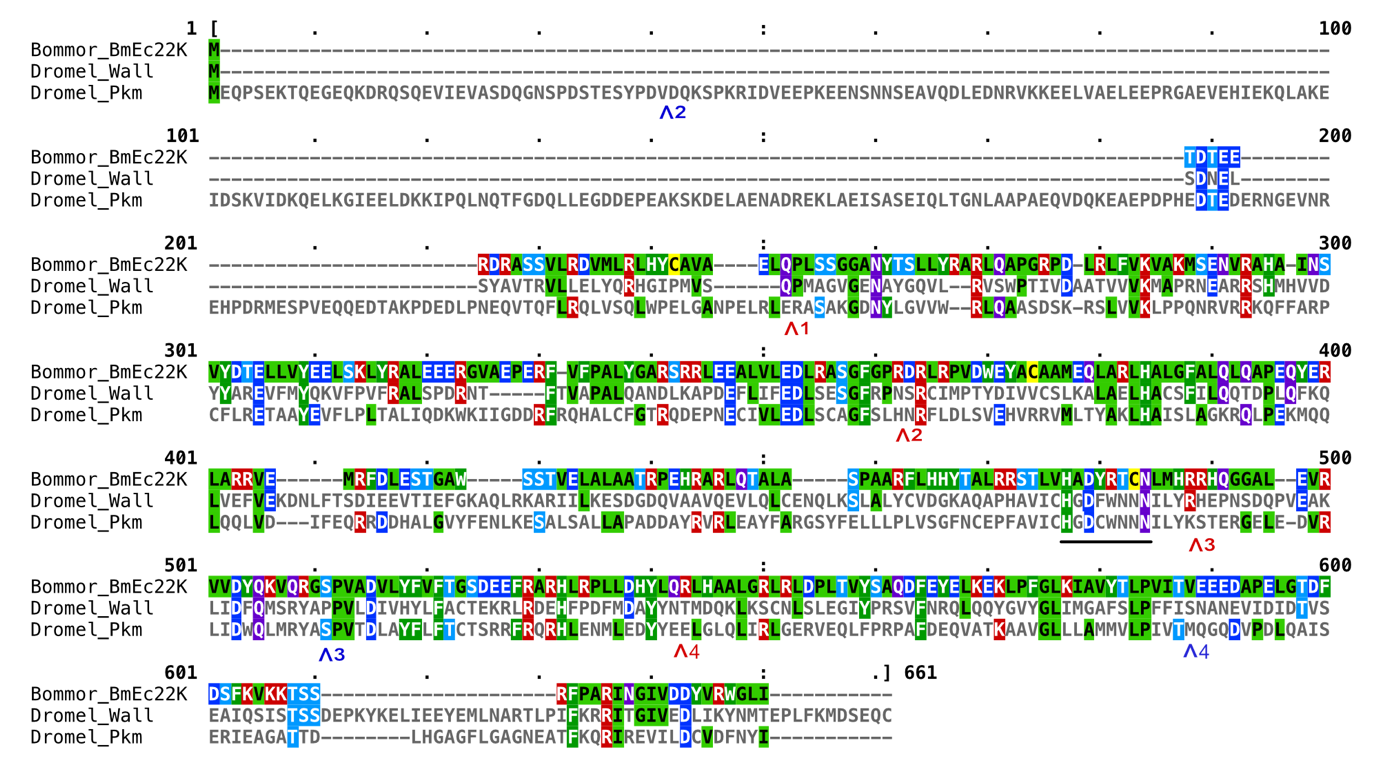


**Figure S2.** Amino acid sequence alignment of *BmEc22K*, *Wall* and *pkm*. Residues with identity to *BmEc22K* are coloured. The black underline is the location of the ATP-binding Brenner’s motif (HxDxxxxN). Red and blue carets and numbers (eg. ^1) indicate the expected cleavage locations for the CRISPR gRNAs for *Wall* and *pkm*, respectively. Note: gRNA1 for *pkm* binds upstream of the start codon and is not shown. Sequence alignment was generated with MAFFT v7.525 (Katoh and Standley 2013) and visualised with MView (Brown *et al.* 1998).

**
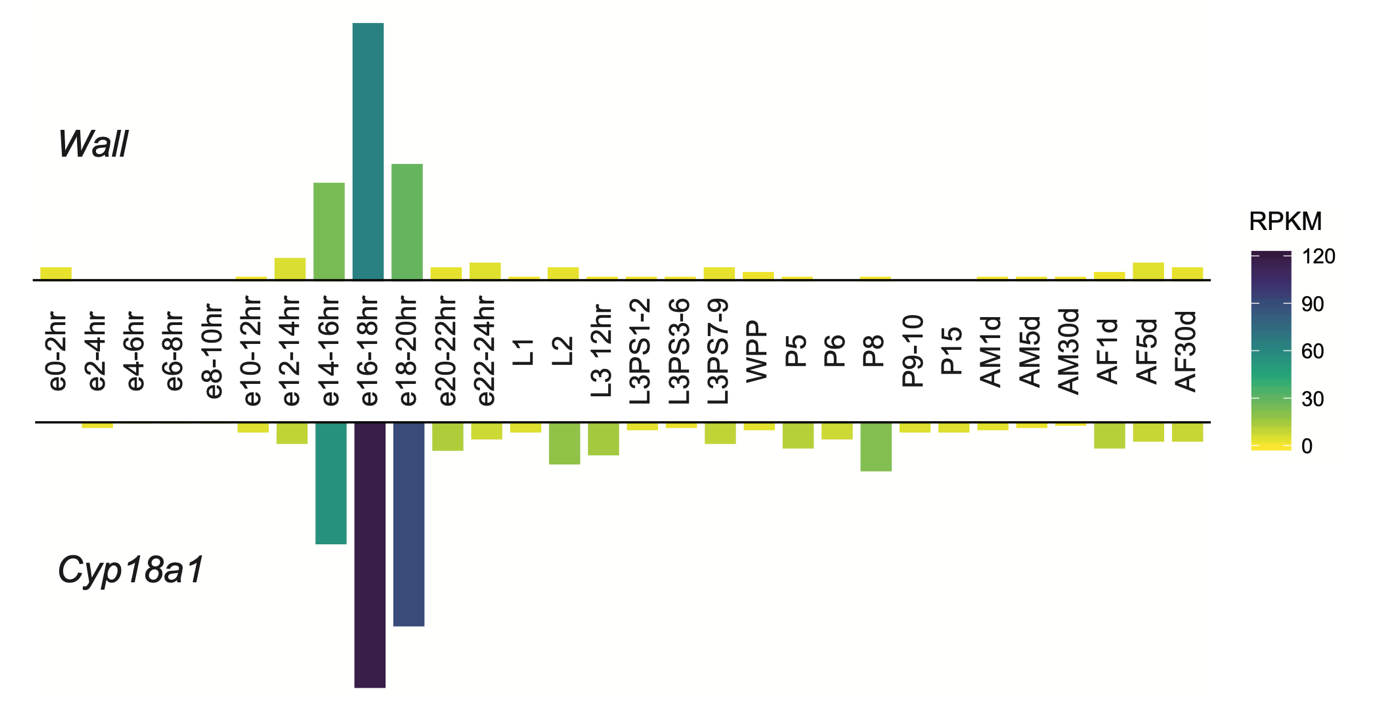
**

**Figure S3** **.** Expression of *Wall* and *Cyp18a1* throughout *Drosophila melanogaster* development, based on data from modENCODE (Graveley *et al.* 2011). Gene expression is given in RPKM (Reads Per Kilobase of transcript, per Million mapped reads) mapped onto the colour of each bar, while the height of the bars for each gene is normalised to the highest level of expression for that gene. e, embryo (in hrs after fertilisation); L1, 1^st^ instar larva; L2, 2^nd^ instar larva; L3, 3^rd^ instar larva (12 hr after moulting and the salivary gland puff stages); WPP, white prepupa; P, pupa (stages from Bainbridge and Bownes 1981); AM, adult male (1–30 days post-eclosion); AF adult female (1–30 days post-eclosion).

**
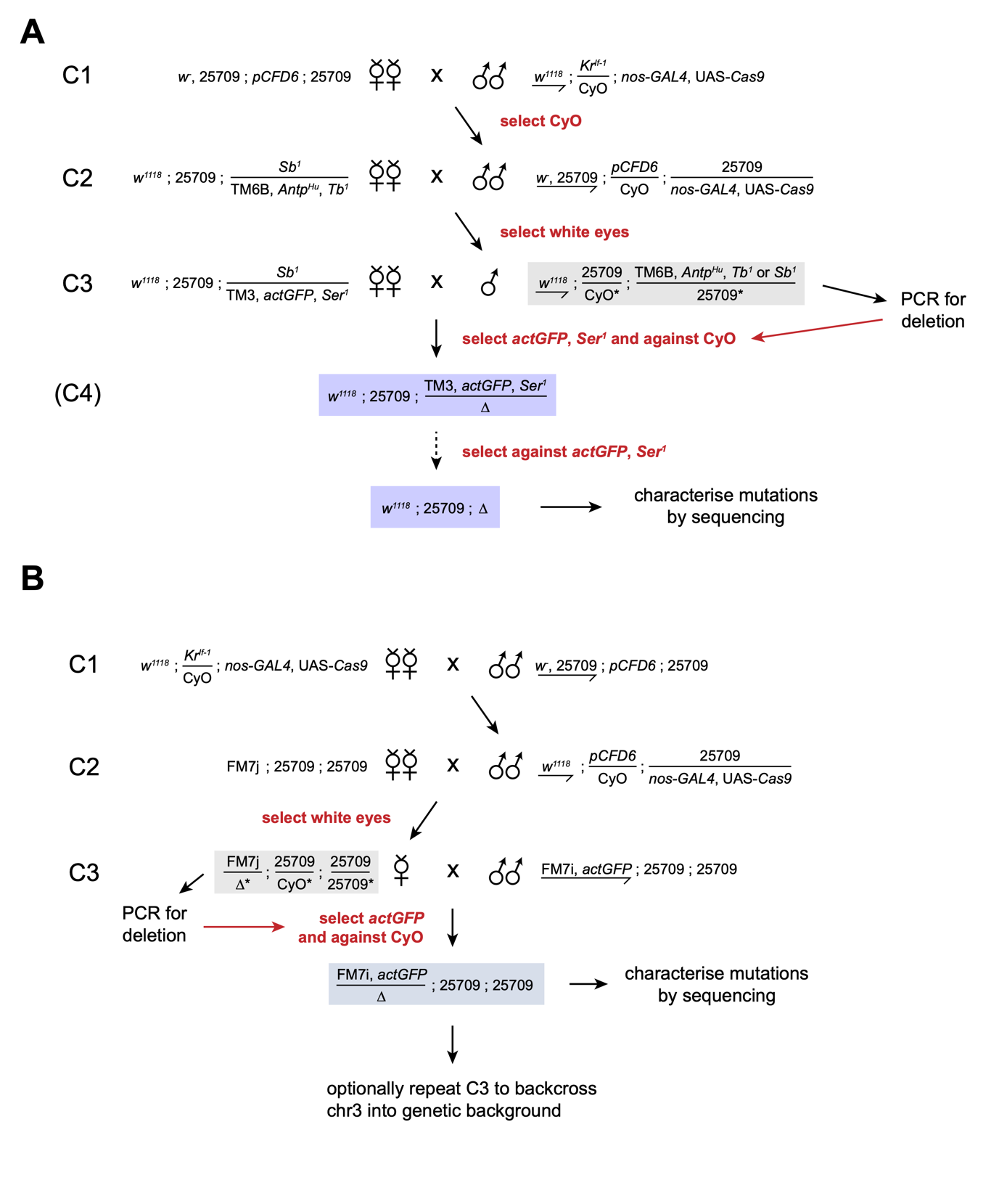
**

**Figure S4** **.** CRISPR-Cas9 mutagenesis crossing schemes. (A) Crossing scheme for mutagenesis of a wild-type chr3 locus in the BL25709 genetic background to produce a deletion allele (∆). The single males used in C3 (grey box) are ‘founder males’ of each potential mutant line. Males used in C3 are selected by the colour of their eyes—as the *pCFD6*, *nos-GAL4* and UAS-*Cas9* constructs all contain a mini-white gene (*w^+mC^*) that produces orange eyes in a *w^-^* background, individuals that inherit all three transgenic constructs (i.e. mutagenic males) can be distinguished from those that only inherit only two (*nos-GAL4* and UAS-*Cas9*). Dashed arrows indicate a possible homozygosing step (if the alleles generated are homozygous-viable). C3 can use either TM3, *actGFP*, *Ser^1^* or *Sb^1^* males as founders, in order to double the number of potential mutant lines that can be generated from the cross.

(B) Crossing scheme for CRISPR-Cas9 mutagenesis on the X chromosome (chrX) using *pCFD6*-transformed (UAS-4xgRNA) flies, to produce a deletion allele (∆) in a final line (grey-blue box) that can be homozygosed if possible. The single females used in C3 (grey box) are ‘founder females’ of each potential mutant line. Asterisks indicate potentially on-target or off-target mutagenised chromosomes.

**
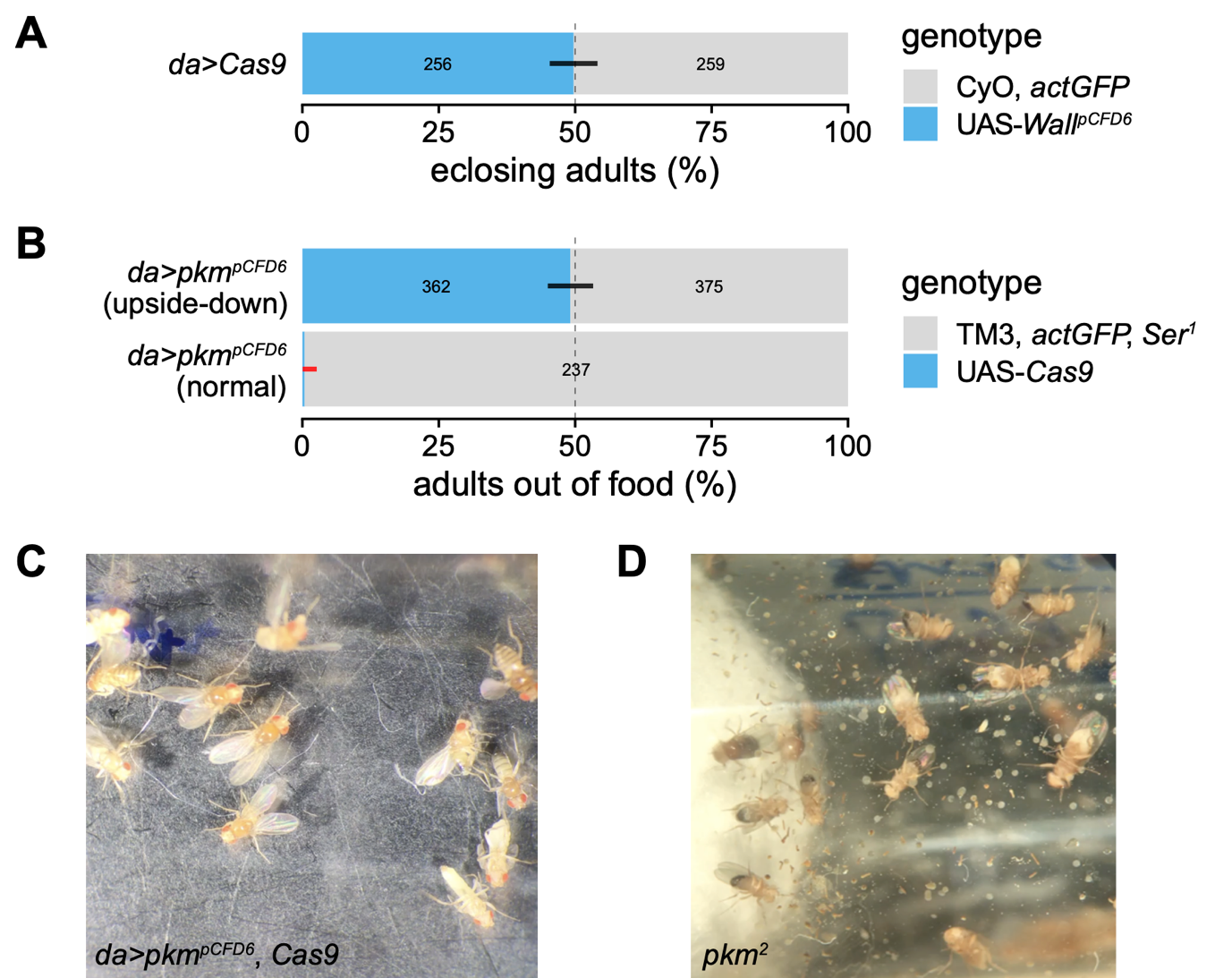
**

**Figure S5** **.** Somatic CRISPR-Cas9 mutagenesis. (A–B) Egg-to-adult viability of ubiquitous somatic CRISPR-Cas9 mutagenesis of (A) *Wall* and (B) *pkm* at 25 ºC, estimated from the adult genotypic ratios of offspring from crosses between (A) *da-GAL4* females and UAS-*Wall^pCFD6^*/CyO *actGFP*; UAS-*Cas9* males or (B) *da-GAL4* females and UAS-*pkm^pCFD6^*; UAS-*Cas9*/TM3 *actGFP* *Ser^1^* males. The dashed line indicates the expected 1:1 genotypic ratio if both genotypes per cross are equally developmentally viable; black and red error bars indicate non-significant or significant deviations, respectively, from expected genotypic ratios after correction for multiple tests. Numbers on the bars are the number of adults of each genotype (for numbers greater than one). (A) Error bars are 95% confidence intervals for the proportion of UAS-*Wall^pCFD6^*-containing (somatic knockout) heterozygotes. Vials were held in the normal orientation throughout development. (B) Error bars are 97.5% confidence intervals (95% CI adjusted for two tests) for the proportion of UAS-*Cas9*-containing (somatic knockout) heterozygotes. Vials were kept in their normal orientation after pupation (‘normal’) or tipped upside-down (‘upside-down’), to check if pre-adult development is affected by somatic knockout. (C) Non-motile but otherwise viable *da>pkm^pCFD6^* *Cas9* adults, rescued from ‘drowning’ in food substrate by eclosing in vials tipped upside-down, viewed on the bottom side of a vial resting on its side; note the ‘held-out’ wing posture of many flies. (D) Motile, viable *pkm^2^* adults, viewed on the top side of a vial resting on its side.

**
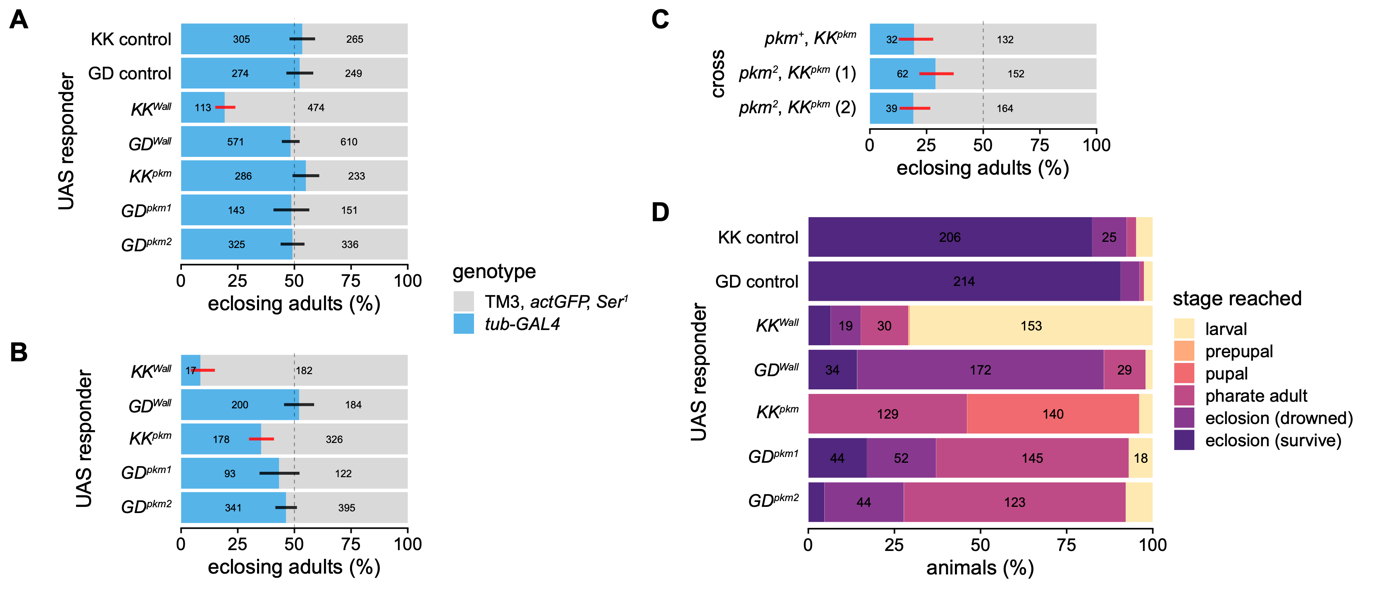
**

**Figure S6** **.** RNAi knockdown of *Wall* and *pkm*. (A–C) Egg-to-adult viability of RNAi knockdown (or control genotypes), estimated from the adult genotypic ratios of offspring from crosses between (A–B) *tub-GAL4*/TM3 *actGFP* *Ser^1^* females and UAS-responder (or control genetic backgrounds) males, or (C) UAS-responder females and *tub-GAL4*/TM3 *actGFP* *Ser^1^* males. The dashed line indicates the expected 1:1 genotypic ratio if both genotypes per cross are equally developmentally viable; black and red error bars indicate non-significant or significant deviations, respectively, from expected genotypic ratios after correction for multiple tests. Numbers on the bars are the number of adults of each genotype (for numbers greater than 0). KK control genotype is VL60100, GD control genotype is VL60000. (A) Knockdown crosses conducted at 25 ºC. Error bars are 99.3% confidence intervals (95% CI adjusted for seven tests) for the proportion of *tub-GAL4*-containing heterozygotes. Data from the KK control, GD control and *KK^pkm^* crosses are from Scanlan et al. (2020) but were collected together with the others shown here. (B) Knockdown crosses conducted at 29 ºC. Error bars are 99% confidence intervals (95% CI adjusted for five tests) for the proportion of *tub-GAL4*-containing heterozygotes. (C) Knockdown crosses conducted at 29 ºC to test for genetic compensation (lack of knockdown phenotype in a null mutant background). Maternal genotypes were *pkm^+^*; KK*^pkm^* (VL106503; top), and two independently generated *pkm^2^*; KK*^pkm^* lines (middle and bottom). Error bars are 98.3% confidence intervals (95% CI adjusted for three tests) for the proportion of *tub-GAL4*-containing heterozygotes. (D) Larval-to-adult viability of GFP-negative offspring from crosses between UAS-*Dcr2*/CyO *actGFP*; *tub-GAL4*/TM3 *actGFP* *Ser^1^* females and UAS-responder (or control genetic backgrounds) males. Numbers on the bars are the number of individuals in each lethal phase category (for numbers greater than 15).

**
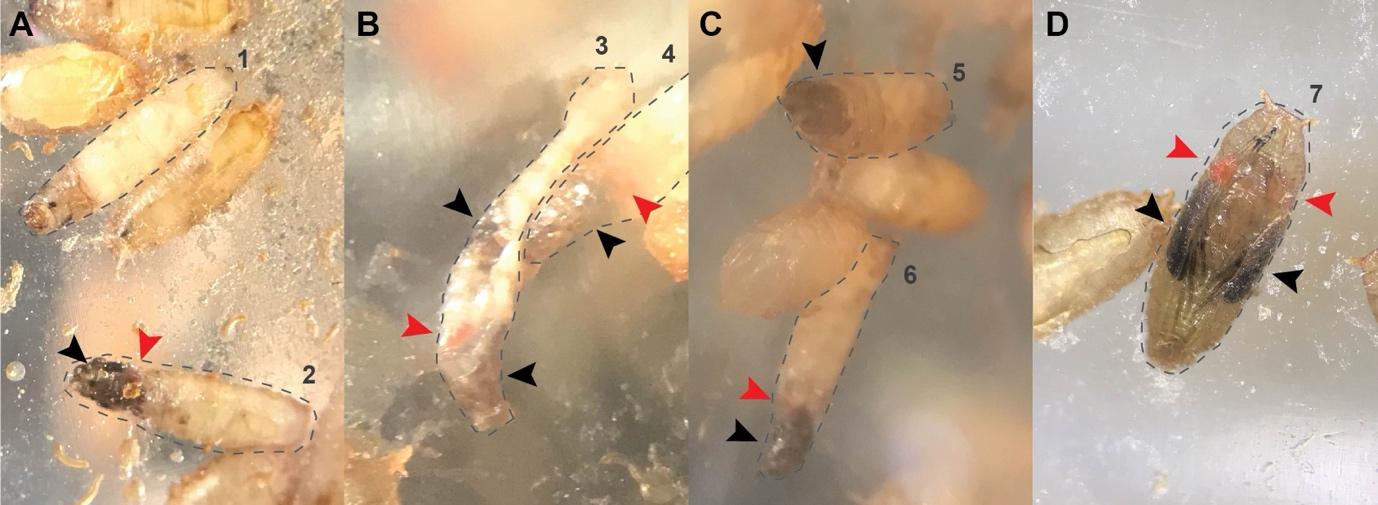
**

**Figure S7** **.** (A–C) Phenotype of *HR>Wall^EY^* animals at 25 ºC (photos also contain some non-misexpression animals). Grey dashed lines separate the visible regions of each misexpression animal of note, which are also numbered (1–6). Regions of dark (thoracic/wing) pigmentation are marked with black arrowheads, regions of red (eye) pigmentation are marked with red arrowheads. (A) Animal 1 shows an early stage of the phenotype, before the differentiation of adult structures within the puparium. Animal 2 shows a later stage, where the adult thorax and head have differentiated without head eversion, resulting in red eye pigment posterior to the dark pigment of the wings. (B) Similar phenotypes to animal 2 in animals 3 and 4, although 3 has two regions of dark pigmentation. Note that animal 3 appears ‘deflated’, which was common in older animals, suggestive of cuticular defects. (C) Animal 5 is viewed mostly from a superior perspective, obscuring the red pigment, while animal 6 is viewed along the length of the body, again revealing red pigment posterior to the dark pigment. (D) Wild-type animal (7) showing normal pharate adult development and the positions of the eyes and the wings. **
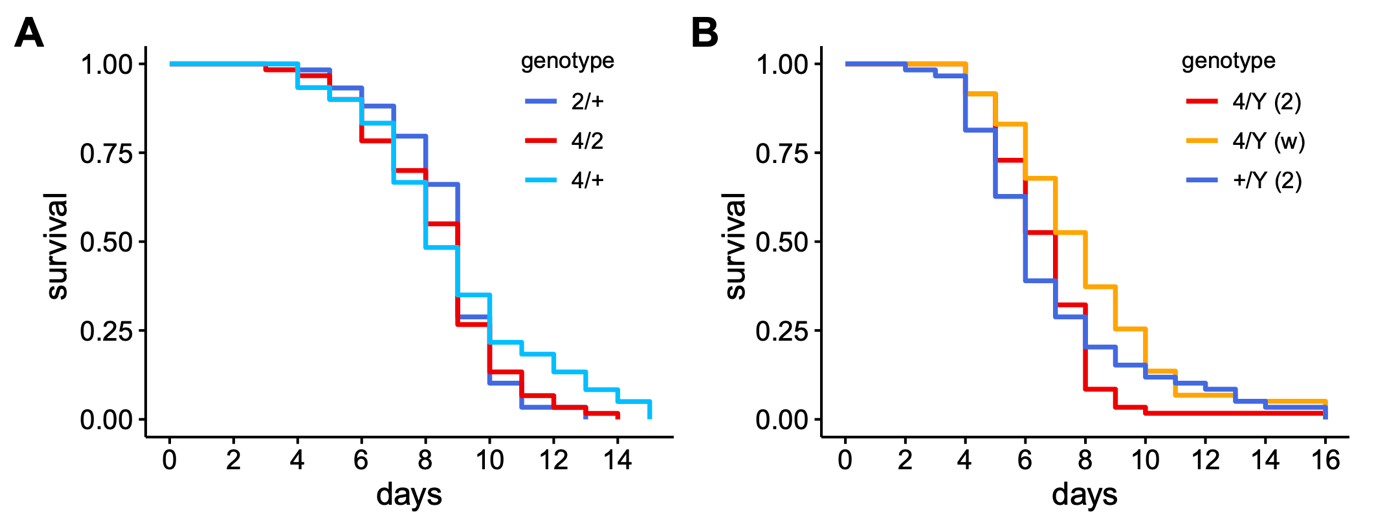
**

**Figure S8.** Wet-starvation survival experiments with *pkm* loss-of-function mutants (red and orange) and wild-type (dark blue and light blue) animals. (A) Virgin female survival curves. (B) Virgin male survival curves. Genotypes: 2/+ = *pkm^2^*/*pkm^+^*; 4/2 = *pkm^4^*/*pkm^2^*; 4/+ = *pkm^4^*/*pkm^+^*; 4/Y (2) = *pkm^4^*/Y (*pkm^2^* father); 4/Y (w) =*pkm^4^*/Y (*w^1118^* father); +/Y (2) = *pkm^+^*/Y (*pkm^2^* father). n = 59–60 adults per genotype. **
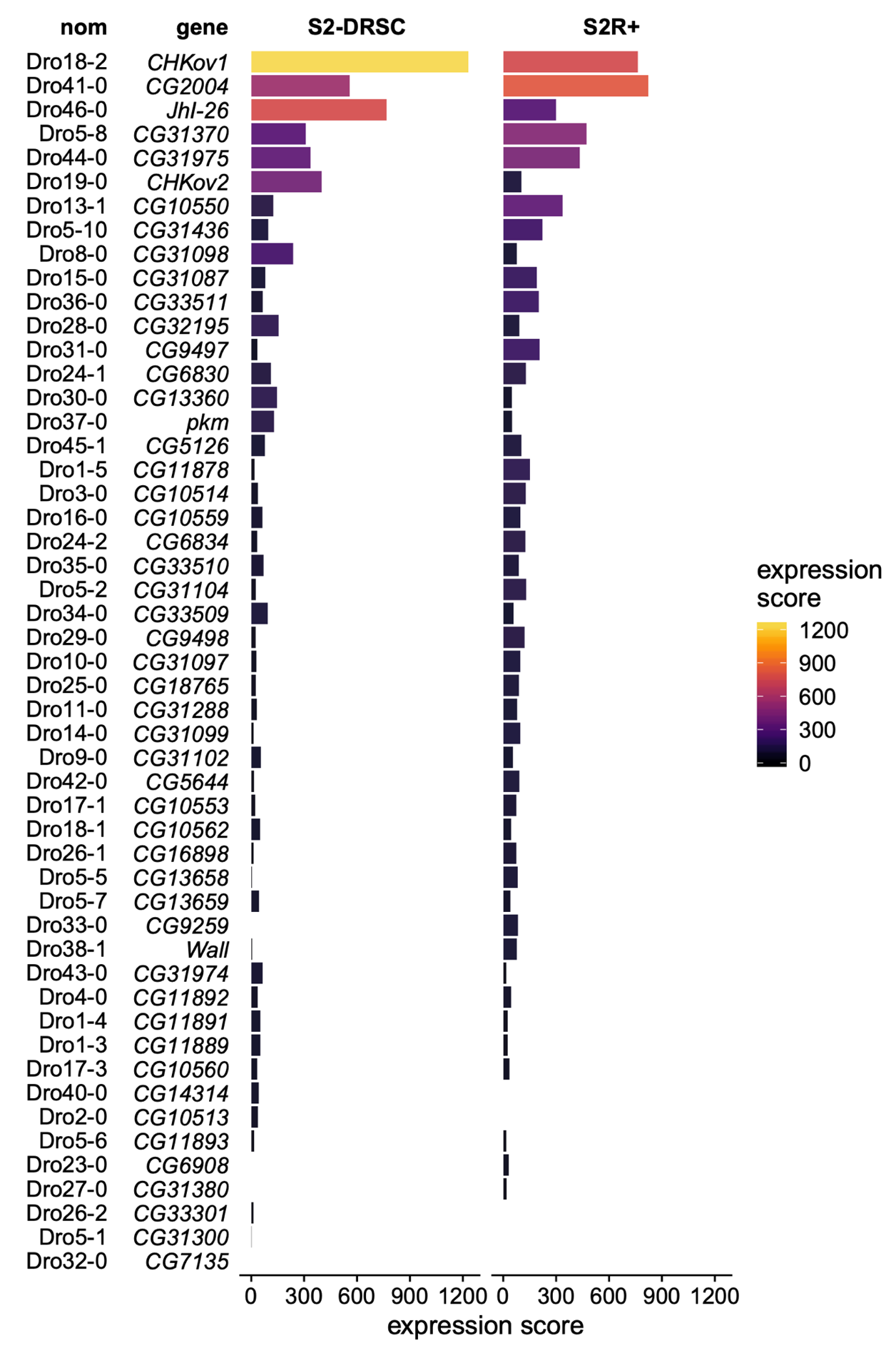
**

**Figure S9** **.** Basal expression (‘expression scores’ from microarrays) of *D. melanogaster* EcKL genes in two isolates of the S2 cell line—S2-DGRC and S2R+—as reported by Cherbas et al. (2011). Genes are ordered by combined total expression score. nom, *Drosophila* genus EcKL nomenclature (Scanlan *et al.* 2020).**Supplementary References**

Bainbridge, S. P., and M. Bownes, 1981 Staging the metamorphosis of Drosophila melanogaster. J. Embryol. Exp. Morphol. 66: 57–80.

Brown, N. P., C. Leroy, and C. Sander, 1998 MView: a web-compatible database search or multiple alignment viewer. Bioinformatics 14: 380–381.

Cherbas, L., A. Willingham, D. Zhang, L. Yang, Y. Zou *et al.*, 2011 The transcriptional diversity of 25 Drosophila cell lines. Genome Res. 21: 301–314.

Graveley, B. R., A. N. Brooks, J. W. Carlson, M. O. Duff, J. M. Landolin *et al.*, 2011 The developmental transcriptome of Drosophila melanogaster. Nature 471: 473–479.

Green, E. W., G. Fedele, F. Giorgini, and C. P. Kyriacou, 2014 A Drosophila RNAi collection is subject to dominant phenotypic effects. Nat. Methods 11: 222–223.

Hoang, D. T., O. Chernomor, A. von Haeseler, B. Q. Minh, and L. S. Vinh, 2018 UFBoot2: Improving the Ultrafast Bootstrap Approximation. Mol. Biol. Evol. 35: 518–522.

Katoh, K., and D. M. Standley, 2013 MAFFT Multiple Sequence Alignment Software Version 7: Improvements in Performance and Usability. Mol. Biol. Evol. 30: 772–780.

Scanlan, J. L., P. Battlay, and C. Robin, 2022 Ecdysteroid kinase-like (EcKL) paralogs confer developmental tolerance to caffeine in Drosophila melanogaster. Current Research in Insect Science 2: 100030.

Scanlan, J. L., R. S. Gledhill-Smith, P. Battlay, and C. Robin, 2020 Genomic and transcriptomic analyses in Drosophila suggest that the ecdysteroid kinase-like (EcKL) gene family encodes the ‘detoxification-by-phosphorylation’enzymes of insects. Insect Biochem. Mol. Biol. 123: 103429.

Scanlan, J. L., and C. Robin, 2024 Phylogenomics of the Ecdysteroid Kinase-like (EcKL) Gene Family in Insects Highlights Roles in Both Steroid Hormone Metabolism and Detoxification. Genome Biol. Evol. 16:.
